# Targeting of Beta Adrenergic Receptors Results in Therapeutic Efficacy against Models of Hemangioendothelioma and Angiosarcoma

**DOI:** 10.1371/journal.pone.0060021

**Published:** 2013-03-28

**Authors:** Jessica M. Stiles, Clarissa Amaya, Steven Rains, Dolores Diaz, Robert Pham, James Battiste, Jaime F. Modiano, Victor Kokta, Laura E. Boucheron, Dianne C. Mitchell, Brad A. Bryan

**Affiliations:** 1 Department of Biomedical Sciences, Paul L. Foster School of Medicine, Texas Tech University Health Sciences Center, El Paso, Texas, United States of America; 2 Klipsch School of Electrical and Computer Engineering, New Mexico State University, Las Cruces, New Mexico, United States of America; 3 Department of Neurology, University of Texas Southwestern Medical Center, Dallas, Texas, United States of America; 4 Masonic Cancer Center, Department of Veterinary Clinical Sciences, University of Minnesota, Minneapolis, Minnesota, United States of America; 5 Department of Pathology, CHU Sainte-Justine, University of Montreal, Montreal, Quebec, Canada; Virginia Commonwealth University, United States of America

## Abstract

Therapeutic targeting of the beta-adrenergic receptors has recently shown remarkable efficacy in the treatment of benign vascular tumors such as infantile hemangiomas. As infantile hemangiomas are reported to express high levels of beta adrenergic receptors, we examined the expression of these receptors on more aggressive vascular tumors such as hemangioendotheliomas and angiosarcomas, revealing beta 1, 2, and 3 receptors were indeed present and therefore aggressive vascular tumors may similarly show increased susceptibility to the inhibitory effects of beta blockade. Using a panel of hemangioendothelioma and angiosarcoma cell lines, we demonstrate that beta adrenergic inhibition blocks cell proliferation and induces apoptosis in a dose dependent manner. Beta blockade is selective for vascular tumor cells over normal endothelial cells and synergistically effective when combined with standard chemotherapeutic or cytotoxic agents. We demonstrate that inhibition of beta adrenergic signaling induces large scale changes in the global gene expression patterns of vascular tumors, including alterations in the expression of established cell cycle and apoptotic regulators. Using *in vivo* tumor models we demonstrate that beta blockade shows remarkable efficacy as a single agent in reducing the growth of angiosarcoma tumors. In summary, these experiments demonstrate the selective cytotoxicity and tumor suppressive ability of beta adrenergic inhibition on malignant vascular tumors and have laid the groundwork for a promising treatment of angiosarcomas in humans.

## Introduction

A wealth of evidence indicates that stressful situations exacerbate tumor progression. Adrenergic processes stimulated by epinephrine and norephinephrine drive the development of tumor growth and metastasis [1], [2], [3]. Breast cancer models indicate the sympathetic nervous system serves as a neural regulator inducing a metastatic switch, with stress-induced neuroendocrine activation leading to a 30-fold increase in tumor metastasis [4]. Moreover, beta adrenergic receptor antagonists have shown efficacy against melanoma [5], [6], breast cancer [7], [8], [9], [10], [11], [12], and prostate cancer [3], and high beta adrenergic receptor expression in tumors is associated with poor clinical outcome in breast cancer patients [13]. One prospective study revealed a 36% reduction in the risk of melanoma progression for each year of beta blocker treatment [6].

The non-specific beta adrenergic receptor inhibitor propranolol has been utilized as the gold standard treatment in pediatric patients with infantile hemangioma, a benign vascular tumor which affects up to 10% of the Caucasian population [14]. Infantile hemangiomas express high levels of beta adrenergic receptors potentially explaining their sensitively to propranolol, and these receptors are reported to be high in malignant vascular tumors [15]. This suggests the efficacy of propranolol and other beta blockers may extend to aggressive vascular tumors. Several reports indicate positive results from beta blockade in patients with moderately threatening vascular tumors including kaposiform hemangioendothelioma, tufted angiomas, parotid hemangioma, Kasabach-Merritt phenomenon, and drug resistant hepatic hemangiomas [16], [17], [18], [19], [20]. It remains to be determined if more malignant vascular tumors such as the rare and understudied metastatic hemangioendotheliomas and angiosarcomas are susceptible to the effects of beta blockade. Such a study is essential given the five year survival rates for angiosarcomas and metastatic hemangioendotheliomas are very poor. Treatment of angiosarcomas typically involves surgery, radiation, and neoadjuvant and/or adjuvant chemotherapy with doxorubicin or taxanes, yet even following aggressive therapy, patient survival in metastatic disease is abysmally low [21], [22]. Hemangioendotheliomas are often associated with low mitotic index and are typically more manageable, however approximately 30% of these tumors display aggressive behavior, rapid disease progression, and eventual patient death despite extensive treatment [23], [24]. Given the lack of adequate therapies for angiosarcomas and aggressive hemangioendotheliomas, there exists a strong need for the development of treatments against these unique tumor types. Based on the remarkable efficacy of propranolol against benign vascular tumors and the recent report that malignant vascular tumors exhibit high beta adrenergic receptor expression we hypothesized that hemangioendotheliomas and angiosarcomas should be particularly susceptible to propranolol. Thus we examined the effects of propranolol on the oncogenic properties of a panel of malignant vascular tumor lines and the growth of *in vivo* angiosarcoma tumors. Our data lay the preclinical groundwork for the future clinical use of beta blockade against malignant vascular tumors in humans.

## Materials and Methods

### Ethics Statement

Use of de-identified human tissues was approved by the Texas Tech University Health Sciences Center Institutional Review Board for the Protection of Human Subjects (IRB E13029). Waiver of informed consent was approved by IRB under 35 CFR 46.117(c)(1). All mouse experimentation was approved by the Texas Tech University IACUC committee (IACUC #12020) and conducted according to responsible guidelines for the care and use of laboratory animals. Animals were sacrificed using CO2 asphyxiation.

### Cell Culture and Reagents

The mouse SVR angiosarcoma line (ATCC #CRL-2280) and EOMA hemangioendothelioma line (ATCC #CRL-2586) were cultured in Dulbecco’s modified Eagle’s medium supplemented with 10% fetal bovine serum (FBS) and penicillin/streptomycin (P/S). The canine angiosarcoma lines Emma, Frog, and SB were cultured as previously reported [25]. Primary cultures of human dermal microvascular endothelial cells (HDMVECs, ATCC #CRL-4025) were grown in vascular cell basal media (ATCC #PCS-100-030) and supplemented with 0.2% bovine brain extract, 5 ng/ml human epidermal growth factor, 10 mM L-glutamine, 0.75 units/ml heparin sulfate, 1 µg/ml hydrocortisone, 50 µg/ml ascorbic acid, 2% fetal bovine serum, and pen/strep. Propranolol was diluted in dimethyl sulfoxide for all *in vitro* experiments or isotonic saline for all *in vivo* experiments. Cytotoxic agents were used at the following concentrations: cisplatin (10 µM), busulfan (80 µM), vincristine (1 µM), and H2O2 (1∶5000 dilution of 3% stock).

### mRNA Expression

For qPCR, RNA from 100 µM propranolol or sham treated SVR cells (24 hours treatment) was purified using the Purelink RNA mini kit (Ambion) and converted to cDNA using the Verso cDNA synthesis kit (Thermo-Scientific). qPCR was performed in triplicate using TaqMan probes for ADRB1 (Invitrogen; human/mouse #Hs02330048_s1, canine #Cf02691262_g1), ADRB2 (Invitrogen; human/mouse #Hs00240532_s1, canine #Cf02690130_s1), and ADRB3 (Invitrogen; human/mouse #Hs00609046_m1, canine #Cf03022965_s1) or qPCR arrays (SABiosciences; apoptosis array #PAMM-012Z, cell cycle array #PAMM-020Z) on an ABI7900HT real time PCR instrument (Applied Biosystems). Microarrays on SVR cells treated for 24 hours with 100 µM propranolol or sham were performed in triplicate from pooled samples of three biological replicates for each condition as previously reported [26]. Briefly, total RNA was isolated from each replicate using the Purelink RNA Micro kit (Invitrogen), pooled, and amplified and biotin-labeled using Illumina TotalPrep RNA Amplification Kit (Ambion). 750 ng of biotinylated aRNA was then briefly heat-denatured and loaded onto expression arrays to hybridize overnight. Following hybridization, arrays were labeled with Cy3-streptavidin and imaged using the Illumina ISCAN. Intensity values were transferred to Agilent GeneSpring GX microarray analysis software and data was filtered based on the quality of each call. Statistical relevance was determined using ANOVA with a Benjamini Hochberg FDR multiple testing correction (p-value <0.05). Data were then limited by fold change analysis to statistically relevant data points demonstrating a 2-fold or greater change in expression. Omics pathway analysis was performed with Metacore integrated software suite (GeneGo). Microarray data was publically deposited in Gene Expression Omnibus (Accession # GSE42534).

### Immunohistochemistry

Immunohistochemical studies were performed on 5 µm thick, formalin fixed, paraffin-embedded angiosarcoma sections obtained from Hospital Ste Jutine (Montreal, Canada). Sections were deparaffinized, rehydrated, and treated for antigen retrieval using Trilogy (Cell Marque; Cat. No. 920P-10). To block nonspecific binding, sections were incubated in Background Block solution (Cell Marque; Cat. No. 927B-05) at room temperature for 10 minutes before application of the primary antibody. The following primary antibodies used were: rabbit anti-ADBR1 (1∶200, Abbiotec Cat. No. 250919), rabbit anti-ADBR2 (1∶200, Abbiotec Cat. No. 251604), and rabbit anti-ADBR3 (1∶200, Abbiotec Cat. No.251434). Sections were then washed in PBS IHC Wash Buffer with tween 20 (Cell Marque; Cat. No. 934B-09) three times for 5 minutes each and then incubated with the CytoScan Alkaline Phos Detection System (Cell Marque; Cat. No. 952D-20). The immunostaining was carried out using Permanent Red Chromogen detection kit (Cell Marque; Cat. No. 960D-20) and counterstained with Hematoxylin.

### Cell Proliferation/survival

Cells were plated at approximately 50% confluence and treated with a dose curve of 0 to 200 µM propranolol over a 48 hour time course. Time lapse microscopy was performed using a BioStation CT (Nikon) and the change in cell number over the experimental time course was determined by manually counting cells per vision field. For growth in three dimensional culture to examine the effects of chronic low doses of propranolol on vascular tumor cell survival, cells were seeded onto Alvetex membranes at 2×10^5^ cells per (12) well, allowed to establish for 48 hours, and subsequently treated with a sub-lethal concentration of propranolol (20 µM) or sham over a period of 6 days, after which the membranes were stained with Hoechst 33342 to label the nuclei to facilitate cell counting. Numerical data presented for all proliferation experiments is the average of at least three biological replicates +/− the standard deviation. Statistical significance was determined using Student’s t-test (P<0.05).

### Immunofluorescence

Cells/tissues were fixed with 4% paraformaldehyde, permeabilized in 0.01% Triton X-100, blocked in 5% milk plus 0.05% Tween-20, and incubated with antibodies against either p21 (1∶50, Abcam #ab7960), p27 (1∶200, Cell Signaling #3698), cleaved caspase 3 (1∶100, Cell Signaling #9664), or proliferating cell nuclear antigen (1∶2500, Cell Signaling #2586). For actin cytoskeletal detection, cells were incubated with rhodamine-labelled phalloidin (1∶150, Cytoskeleton Inc.). The primary antibodies were then detected with Alexa Fluor secondary antibodies (Invitrogen). Cells were counterstained with Hoechst 33342 and imaged on a Nikon C2SI laser scanning confocal microscope. For immunoflourescent detection of the apoptotic index, cells were stained for 10 minutes with 5 µg/ml Hoechst 33342 and 5 µg/ml propidium iodide, and washed 3× in phosphate buffered saline. Images of nuclei from cells treated with 100 µM propranolol for 24 hrs were equivalently processed in Nikon Elements 3.2, surface rendering images were obtained using Imaris 6.0, and 3D deconvolution was performed using Autoquant X3.

### Western Blotting

Cell lysates were collected after 1 or 24 hours treatment (as indicated in the results), subjected to SDS-PAGE, and transferred to nitrocellulose membranes using the Trans-Blot Turbo Transfer System (Biorad). Membranes were incubated with antibodies obtained from Cell Signaling [phospho-p38 (1∶1000, #4511), p38 (1∶1000, #8690), Cdk4 (1∶1000, #2906), Cdk6 (1∶1000, #3136), CycD1 (1∶500, #2978), CycE (1∶5000, #4129), PCNA (1∶2500, #2586) “p27 (1∶1000, #3698)” p53 (1∶1000, #2527), cleaved caspase-3 (1∶200, #9664), cleaved PARP (1∶200, #9544), p-cofilin (1∶1000, #3313), p-ERM (1∶1000, #3142), p-MYPT (1∶1000, #3040), or β-actin antibodies (1∶1000, #4970)] or Abcam [p21 (1∶100, #ab7960) p-Tie2 (1∶1000, #ab788142), Tie2 (1∶1000, #ab24859)] followed with 1∶1000 HRP-conjugated secondary. Proteins were detected using Supersignal West Dura Extended Duration Substrate (Thermo Scientific) and digitally captured using a GE Image Quant LAS4000 imaging system.

### Cell Cycle Analysis

Cells treated for 24 hours with 50 µM propranolol were trypsinized, fixed in a 70∶30 ratio of ethanol:phosphate buffered saline overnight, stained with 200 µg/ml ethidium bromide plus 20 µg/ml RNase A, and incubated overnight. DNA content of each cell treatment condition was analyzed using an Accuri C6 flow cytometer. Quantitative analysis of DNA content was performed using CFlow Plus software (Accuri). Quantification of the percentage of cells in each cell cycle phase represents the average of at least three biological replicates +/− the standard deviation. Statistical significance was determined using Student’s t-test (P<0.05).

### Coculture Assays

Primary cultures of human dermal microvascular endothelial cells (HDMVECs, ATCC #CRL-4025) were labeled with Cell Tracker Blue (Invitrogen) and mixed with unlabeled SVR angiosarcoma cells. The coculture was grown in vascular cell basal media (ATCC #PCS-100-030) and supplemented with 0.2% bovine brain extract, 5 ng/ml human epidermal growth factor, 10 mM L-glutamine, 0.75 units/ml heparin sulfate, 1 µg/ml hydrocortisone, 50 µg/ml ascorbic acid, 2% fetal bovine serum, and pen/strep. Cells were treated 48 hours with 100 µM propranolol or sham and Cell Tracker Blue fluorescence (marking the HDMVECs) and phase contrast (visualizing both HDMVECs and SVR cells) were imaged using a Floid Cell Imaging Station (Life Technologies).

### Migration Assay

For scratch assays, confluent cultures of vascular tumor cells were manually scratch wounded with a P-200 micropipette tip, treated with 50 µM propranolol, and wound closure was monitored hourly using a Nikon Biostation CT time lapse imaging robot over an 18 hour period. For the spheroid migration assay, 2000 cells from the Frog and Emma cell lines were cultured in a hanging drop of their growth media (30 µl total volume) for 24 hours and then allowed to adhere to a standard tissue culture substrate in the presence of 50 µM propranolol or sham. Images were obtained 24 hours after attachment using a Nikon TS100 inverted microscope. Data presented for migration experiments is the average of at least three biological replicates +/− the standard deviation. Statistical significance was determined using Student’s t-test (P<0.05).

### Computational Cytoskeleton Analysis

Median stress fiber length was calculated using the FiberScore algorithm [27] for actin cytoskeleton immunofluoresence images from propranolol or sham treated malignant vascular tumor cells (obtained as described in *Immunofluorescence* methods described above). At least 10 images of each condition were used for the analysis.

### *In vivo* Tumor Assays

SVR chorioallantoic membrane (CAM) tumors were grown using the gelatin sponge-chorioallantoic membrane (CAM) assay as previously described [28]. An isotonic saline sham (N = 4 eggs) or 100 µM propranolol (N = 3 eggs) was added daily directly onto the tumors every two days, and tumors were collected 6 days post implantation. Mouse angiosarcoma tumors were generated by subcutaneous injection of 1×10^6^ SVR cells into the dorsolateral flank of 4 week old female mice as previously described [29]. Mice were treated with sham (isotonic saline; N = 15) or propranolol (10 mg/ml; N = 17) via intraperitoneal injections every 2 days. When the sham tumors reached approximately 1 cm in diameter, the tumors were collected. Numerical data presented for all tumor experiments is the average of at least three biological replicates +/− the standard deviation (for the CAM assays) or the standard error of the mean (for the mouse angiosarcoma tumor assays). Statistical significance was determined using Student’s t-test (P<0.05).

## Results

### Beta Adrenergic Receptors are Expressed in Malignant Vascular Tumors

**Figure 1:**
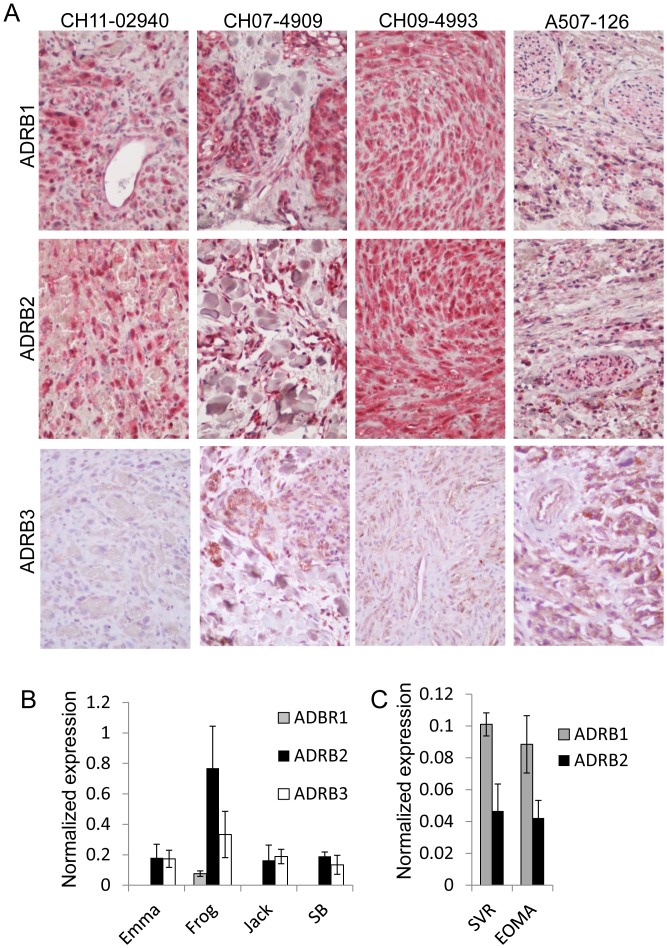
Beta adrenergic receptor expression in malignant vascular tumors. (**A**) Alkaline phosphatase detection of ADRB1, ADRB2, and ADRB3 protein (*red*) in H&E stained human angiosarcoma paraffin tissue sections. (**B & C**) qPCR detection of ADRB1, ADRB2, and ADRB3 mRNA in a panel of canine (**B**) and mouse (**C**) malignant vascular tumor lines Data is provided as the average gene expression +/− standard deviation of at least triplicate biological replicates for each gene.

Beta adrenergic receptors 1, 2, and 3 have been shown to be expressed across a diverse panel of vascular tumors, with the highest expression in malignant vascular tumors [15]. To confirm this, we performed immunohistochemistry for the steady state protein levels of ADRB1, ADRB2, and ADRB3 on four clinically characterized human angiosarcoma tumors, revealing strong signals for ADRB1 and ADRB2 and weaker sample-dependent signals for ADRB3 **(** **Figure 1A** **)**. The clinical data from each angiosarcoma tissue sample is detailed in **Table 1**. We then examined the steady state mRNA expression levels of *ADRB1*, *ADRB2*, and *ADRB3* in a panel of malignant vascular tumor lines including 4 primary canine angiosarcoma cell lines (Emma, Frog, Jack, and SB), one mouse angiosarcoma cell line (SVR), and one mouse hemangioendothelioma cell line (EOMA), demonstrating that canine angiosarcoma lines preferentially express *ADRB2* and *ADRB3* **(** **Figure 1B** **)**, while malignant mouse vascular tumor lines express *ADRB1* and *ADRB2* **(** **Figure 1C** **)**. As infantile hemangiomas (which strongly express beta adrenergic receptors [15], [26]), are clinically susceptible to propranolol, we sought to determine if malignant vascular tumors respond similarly.

**Table 1:** Clinical data for angiosarcoma tissues.

	Histological type	Tumor location	Patient age
CH11-02940	Hemangiosarcoma	Facial	10
CH07-4909	Lymphangiosarcoma	Facial	65
CH09-4993	Hemangiosarcoma	Thigh	8
A507-126	Hemangiosarcoma	Unknown	6

### Propranolol Inhibits Proliferation of Malignant Vascular Tumor Cells in a Dose Dependent Manner

**Figure 2:**
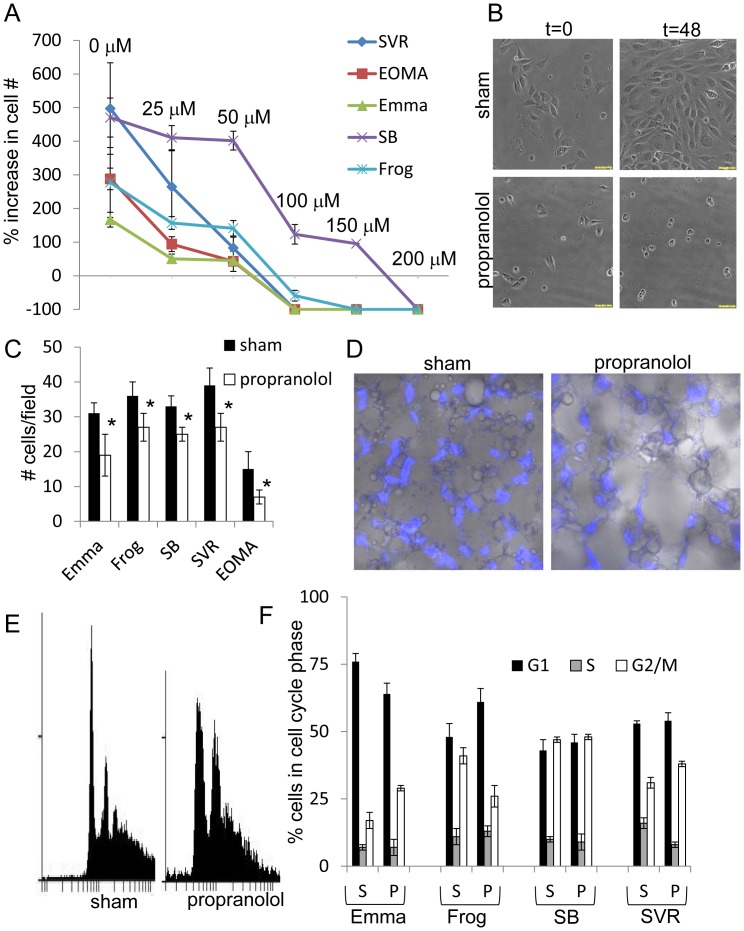
Beta blockade inhibits malignant vascular tumor cell proliferation in a dose dependent manner. (**A**) The panel of malignant vascular tumor lines was subjected to a dose curve of propranolol over a 48 hour time course and changes in cell proliferation were quantified by counting the number of cells per vision field. (**B**) Representative images of sham or 100 µM propranolol treated SVR angiosarcoma cells after 48 hours. (**C**) The panel of malignant vascular tumor lines was grown on Alvetex polystyrene membranes for 48 hours, treated with sham or 25 µM propranolol, and cell density was accessed by quantifying positive Hoechst nuclear staining after 96 hours of treatment. (**D**) Differential interference contast (DIC) and fluorescence image overlays of sham or 25 µM propranolol treated SVR angiosarcoma cells in the Alvetex membranes 96 hours after treatment. (**E**) Cell cycle analysis of propidium iodide stained SVR angiosarcoma cells treated with sham or 100 µM propranolol for 24 hours. (**F**) Cell cycle profile quantification of malignant vascular tumor cells treated with sham or 100 µM propranolol for 24 hours. For all experiments, the data is the average +/− standard deviation for at least three biological replicates. Statistical significant was determined using Students t-test (p<0.05).

To test if propranolol inhibits the proliferation rates of hemangioendothelioma and angiosarcoma cells, we subjected a panel of canine angiosarcoma lines (Emma, SB, and Frog), the mouse angiosarcoma line (SVR), and the mouse hemangioendothelioma line (EOMA) to a dose curve of propranolol for 48 hours and measured cell numbers via time lapse imaging. Propranolol inhibits the proliferation of all malignant vascular tumor cell lines tested in a dose dependent manner **(** **Figure 2A & B** **)**. Even at the lowest concentration of propranolol (25 µM), we observed a cell type dependent 15% to 67% reduction in proliferation, with EOMA hemangioendothelioma cells being the most sensitive and SB angiosarcoma cells the least sensitive. At higher concentrations (100 to 200 µM) significant (p<0.05) cell death occurred across all tumor lines, with 100% lethality in all vascular tumor lines except SB at 100 µM propranolol. Because acute treatment of monolayer cultures fails to recapitulate the environment experienced by tumor cells, we tested chronic low doses of propranolol on malignant vascular tumor cells seeded onto 200 µm thick 3D microcellular polymer membranes. The panel was treated with a chronic low dose of propranolol (20 µM) or sham over a period of 6 days, after which the membranes were stained with Hoechst 33342 to label the nuclei to facilitate cell counting, revealing that under chronic low-dose conditions, the cell panel exhibited a statistically reduced proliferation rates compared to sham conditions **(** **Figure 2C & D** **)**. We then performed flow cytometric analysis on propidium iodide stained vascular tumor cells which were treated with sham or 50 µM propranolol for 24 hours. We utilized 50 µM propranolol for this experiment since our initial experiments at higher doses (100+ µM propranolol) resulted in a large sub-apoptotic peak for each cell line (*data not shown*) thus complicating analysis of how beta blockade affects cell cycle progression. Due to the heterogeneous cell populations composing the EOMA line and complicating interpretation of the data with numerous peaks, we excluded this line from cell cycle analysis. Both SVR and Emma cell lines exhibited a significant increase of cells in the G2/M phase following propranolol addition, while treatment of Frog led to an accumulation of cells in the G1 phase **(** **Figure 2E & F** **)**. The cell cycle profile of propranolol treated SB cells was not significantly different from the sham SB cells, corroborating its relative resistance to the treatment.

**Figure 3:**
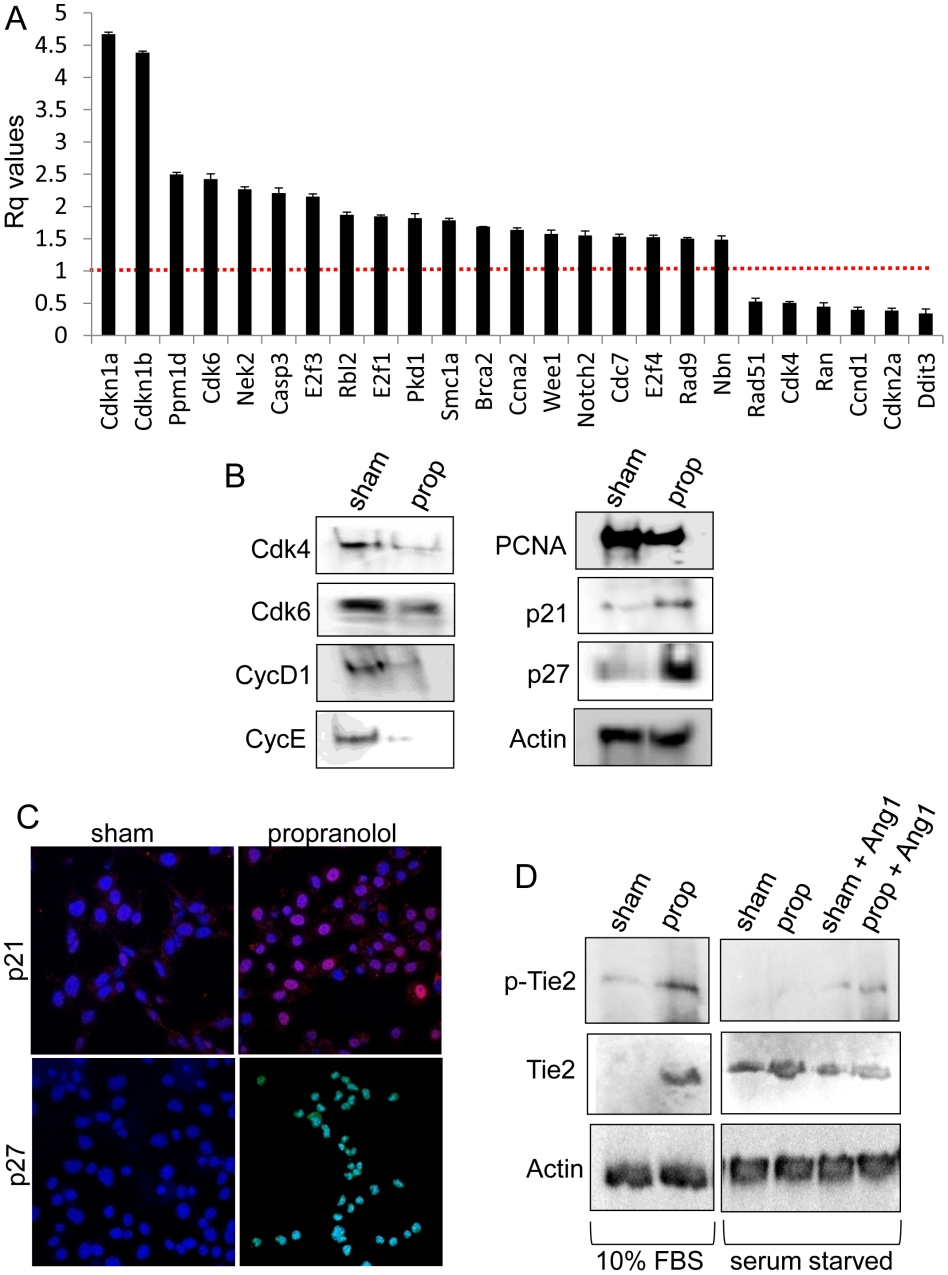
Beta blockade induces alterations in key cell cycle regulators and the Tie2 angiogenic regulator. (**A**) qPCR analysis of a panel of 84 genes involved in cell cycle regulation. Shown are the 25 genes whose mRNA expression levels were statistically altered 1.5 fold or more following 24 hours of 100 µM propranolol in SVR cells. The qPCR data is the average +/− standard deviation for at least three biological replicates. Statistical significant was determined using Students t-test (p<0.05). (**B**) Western blot detecting cell cycle regulatory protein levels in SVR cells subjected to 24 hours of sham or 100 µM propranolol. (**C**) Immunofluorescent staining for p21 (*red*) and p27 (*green*) in SVR cells subjected to 24 hours of sham or 100 µM propranolol. (**D**) SVR cells were grown in standard growth media (10% FBS) or serum starved overnight, treated as indicated with sham, 100 µM propranolol (24 hours), or 10 ng/ml angiopoietin-1 (2.5 minutes), and Western analysis detected the expression of phosphorylated Tie2, total Tie2, and actin.

To identify mechanisms by which propranolol inhibits vascular tumor cell proliferation, we performed a global gene expression microarray on SVR angiosarcoma cells treated with sham or 100 µM propranolol for 24 hours. We identified 84 genes whose expression was statistically altered by more than 3-fold (p<0.05) in response to propranolol and 428 genes exhibiting a significant 2-fold or greater expression alteration. The three fold or greater changes are listed in **Table 2** and the entire dataset can be publically accessed via the Gene Expression Omnibus (Accession # GSE42534). Genego pathway analysis identified five major cellular processes that were altered in response to propranolol including cell cycle, cell survival, cholesterol metabolism, immune response, and cell movement. We followed up on the changes by testing the expression of a number of genes/proteins involved in cell cycle regulation using qPCR arrays on SVR cells treated for 24 hours with sham or 100 µM propranolol. Quantification of the steady state mRNA expression levels of 84 genes known to be involved in cell cycle regulation identified 25 genes whose expression was significantly altered by more than 1.5 fold (p<0.05) in response to propranolol **(** **Figure 3A** **)**. Of particular interest were the 4.7 and 4.4 fold upregulation of the potent cell cycle inhibitors *Cdkn1a* and *Cdkn1b*, respectively. We validated this data via Western and immunofluorescence analysis of *Cdkn1a* and *Cdkn1b* (p21 and p27, respectively) in SVR angiosarcoma lines treated for 24 hours with sham or 100 µM propranolol, revealing significant upregulation of both proteins following propranolol treatment **(** **Figure 3B & C** **)**. Further experimentation revealed decreased steady state protein levels for the proliferative marker PCNA and the key cell cycle regulators Cdk4, Cdk6, cyclin D1, and cyclin E1 **(** **Figure 3C** **)**. Our microarray analysis identified a marked 3.2-fold upregulation in *Tek* expression following 24 hours of propranolol (**Table 2** **)**. This gene encodes for the Tie2 receptor which is a major regulator of vessel maturation and may partly explain the selectivity of propranolol for cells of endothelial origin. 24 hour treatment of SVR cells with 100 µM propranolol resulted in a strong upregulation of Tie2 protein levels, and stimulation of the Tie2 receptor with angiopoietin-1 in serum starved SVR cells treated with either sham or 100 µM propranolol led to a propranolol-dependent increase in Tie2 phosphorylation **(** **Figure 3D** **)**. These data suggest that propranolol may be influencing the transcriptional regulation of signaling pathways involved in vessel quiescence and maturation.

**Table 2:** Three fold or greater changes in gene expression (p<0.05) after 24 hours of 100 mM propranolol in SVR angiosarcoma cells.

Gene Symbol	Gene Name	Accession Number	24 hours
Cryab	Crystallin, alpha B	NM_009964.1	14.8
Slc7a11	Solute carrier family 7, member 11	NM_011990.2	12.7
Hmgcs1	3-hydroxy-3-methylglutaryl-Coenzyme A synthase 1	NM_145942.2	7.9
Fdps	Farnesyl diphosphate synthetase, TV 2,	NM_134469	7.9
2510004L01Rik	Radical S-adenosyl methionine domain containing 2	NM_021384.2	7.7
Acas2	Acyl-CoA synthetase short-chain family member 2	NM_019811.2	6.6
Acat2	Acetyl-Coenzyme A acetyltransferase 2	NM_009338.1	6.3
Hmox1	Heme oxygenase (decycling) 1	NM_010442.1	6.3
Cyp51	Cytochrome P450, family 51	NM_020010	6.1
Slc40a1	Solute carrier family 40 (iron-regulated transporter), member 1	NM_016917.1	6
Gdf15	Growth differentiation factor 15	NM_011819.1	6
Mvd	Mevalonate (diphospho) decarboxylase, TV1	NM_138656.1	5.8
Cxcl1	Chemokine (C-X-C motif) ligand 1	NM_008176.1	5.3
Sc4mol	Sterol-C4-methyl oxidase-like	NM_025436.1	5.3
Gsta3	Glutathione S-transferase, alpha 3, TV2	NM_010356.2	5.1
Pdgfb	Platelet derived growth factor, B polypeptide	NM_011057.2	5
Gsta1	Glutathione S-transferase, alpha 1	NM_008181.2	5
Nqo1	NAD(P)H dehydrogenase, quinone 1	NM_008706.1	4.5
BC031353	cDNA sequence BC031353, TV2	NM_153584.1	4.5
Lss	Lanosterol synthase	NM_146006	4.4
Casp4	Caspase 4, apoptosis-related cysteine peptidase	NM_007609.1	4.3
Gadd45a	Growth arrest and DNA-damage-inducible 45 alpha	NM_007836.1	4
Cd68	CD68 antigen	NM_009853	3.9
Ifit3	Interferon-induced protein with tetratricopeptide repeats 3	NM_010501.1	3.9
Arhgef3	Rho guanine nucleotide exchange factor 3	NM_027871.1	3.9
Idh1	Isocitrate dehydrogenase 1, soluble, TV2	NM_010497	3.9
Klf4	Kruppel-like factor 4	NM_010637.1	3.8
Insig1	Insulin induced gene 1	NM_153526.2	3.8
Cxx1a	CAAX box 1A	NM_024170.1	3.7
Plf2	Prolactin family 2, subfamily c, member 3	NM_011118.1	3.7
Ddit4	DNA-damage-inducible transcript 4	NM_029083.1	3.6
Klhl6	Kelch-like 6	NM_183390.1	3.6
Smad6	SMAD family member 6	NM_008542	3.6
Ypel3	Yippee-like 3	NM_025347.1	3.5
Slc6a9	Solute carrier family 6, member 9	NM_008135	3.5
Clecsf8	C-type lectin domain family 4, member d, TV1	NM_010819.1	3.5
Icam1	Intercellular adhesion molecule 1	NM_010493.2	3.4
Adcy4	Adenylate cyclase 4	NM_080435.1	3.4
Lpin1	Lipin 1, TV1	NM_172950.2	3.4
Hsd17b7	Hydroxysteroid (17-beta) dehydrogenase 7	NM_010476.2	3.4
Stfa3	Stefin A3	XM_147200.1	3.4
2310067E08Rik	Endonuclease domain containing 1	NM_028013.1	3.3
Eltd1	EGF, latrophilin seven transmembrane domain containing 1	NM_133222.1	3.3
Slc9a3r2	Solute carrier family 9, member 3 regulator 2, TVA	NM_023055.1	3.3
9030611O19Rik	Family with sequence similarity 110, member C	NM_027828.2	3.3
Sqstm1	Sequestosome 1	NM_011018.1	3.3
Aacs	Acetoacetyl-CoA synthetase	NM_030210.1	3.2
Tek	Endothelial-specific receptor tyrosine kinase	NM_013690.1	3.2
Oasl1	2'-5' oligoadenylate synthetase-like 1	NM_145209.2	3.2
Atf3	Activating transcription factor 3	NM_007498.2	3.2
BC036718	Nudix (nucleoside diphosphate linked moiety X)-type motif 18	NM_153136	3.2
Slco4a1	Solute carrier organic anion transporter family, member 4a1	NM_148933.1	3.2
Gclm	Glutamate-cysteine ligase, modifier subunit	NM_008129.2	3.2
Adamts4	ADAM metallopeptidase with thrombospondin type 1 motif, 41	NM_172845.1	3.2
Mrpplf3	Prolactin family 2, subfamily c, member 4	NM_011954.2	3.2
Cd40	CD40 antigen (Cd40), TV5	NM_170702.2	3.1
Cxcl10	Chemokine (C-X-C motif) ligand 10	NM_021274.1	3.1
Ikbkg	Inhibitor of kappaB kinase gamma, TV2	NM_178590.2	3.1
Npn3	Sulfiredoxin 1 homolog	NM_029688.2	3.1
Pcyt2	Phosphate cytidylyltransferase 2, ethanolamine	NM_024229.2	3.1
Bhlhb2	Basic helix-loop-helix family, member e40	NM_011498.2	3
Rnf30	Tripartite motif-containing 54	NM_021447.1	3
Hsd3b7	3 beta-hydroxysteroid dehydrogenase type 7	NM_133943.2	3
Nsdhl	NAD(P) dependent steroid dehydrogenase-like	NM_010941.3	3
Brp16	Family with sequence similarity 203, member A	NM_021555.1	−3
Axl	AXL receptor tyrosine kinase (Axl), TV1	NM_009465.2	−3
Lbh	Limb-bud and heart	NM_029999.3	−3.1
Ncl	Nucleolin	NM_010880.2	−3.2
Srm	Spermidine synthase	NM_009272.2	−3.2
Npm3-ps1	Nucleoplasmin 3, pseudogene 1, non-coding RNA	NR_002702.1	−3.4
Npm3	Nucleoplasmin 3	NM_008723.1	−3.8
Hspd1	Heat shock protein 1 (chaperonin)	NM_010477.2	−3.9
Sdpr	Serum deprivation response	NM_138741.1	−3.9
Wnt7b	Wingless-related MMTV integration site 7B, TV1	NM_009528.2	−4
Ankrd1	Ankyrin repeat domain 1 (cardiac muscle)	NM_013468.2	−4.6
Angptl4	Angiopoietin-like 4	NM_020581	−4.9

### Propranolol Induces Apoptosis of Malignant Vascular Tumor Cells

**Figure 4:**
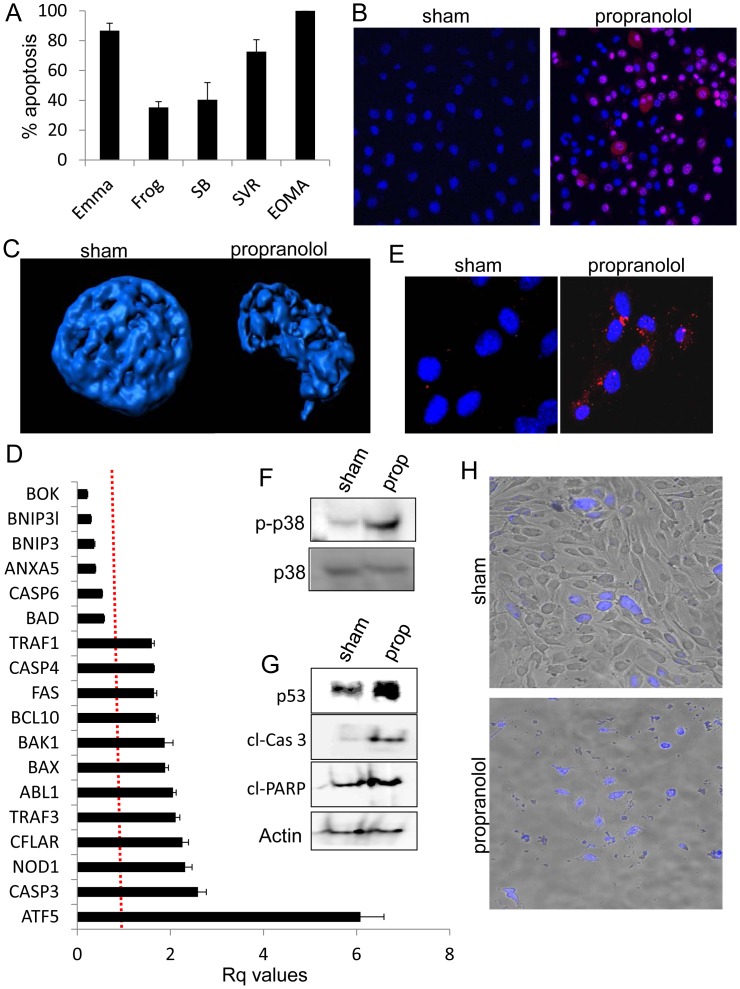
Beta blockade induces apoptosis of malignant vascular tumor cells. (**A**) Apoptosis quantification of Hoechst 33342 and propidium iodide stained malignant vascular tumor cells after 24 hours treatment with sham or 100 µM propranolol. (**B**) Fluorescent images of SVR angiosarcoma cells stained with Hoechst 33342 and propidium iodide after 24 hours treatment with sham or 100 µM propranolol. (**C**) 3D rendering of representative SVR angiosarcoma cell nuclei after 24 hours treatment with sham or 100 µM propranolol. (**D**) qPCR analysis of a panel of 84 genes involved in apoptotic regulation. Shown are the 18 genes whose steady state mRNA expression levels were statistically altered 1.5 fold or more following 24 hours of 100 µM propranolol in SVR cells. (**E**) Immunofluorescent detection of cleaved caspase-3 in SVR angiosarcoma cells after 24 hours treatment with sham or 100 µM propranolol. (**F**) Western analysis detecting phospho-p38 MAPK and total p38 MAPK protein levels after 1 hour of sham or 100 µM propranolol treatment of SVR angiosarcoma cells. (**G**) Western blot detection of apoptotic protein levels. (**H**) Unstained SVR angiosarcoma cells were co-cultured with CellTracker Blue stained HDMVECs. The co-cultures were subjected to sham or 100 µM propranolol and DIC/fluorescent image overlays were obtained after 48 hours treatment. For all experiments, the data is the average +/− standard deviation for at least three biological replicates. Statistical significant was determined using Students t-test (p<0.05).

We observed significant cell death in the panel of malignant vascular tumor cell lines at 100 µM propranolol or greater **(** **Figure 2A** **)**, thus we investigated the contribution of propranolol to hemangioendothelioma and angiosarcoma cell survival. The cell panel was treated with sham or 100 µM propranolol for 24 hours, co-incubated with membrane impermeable propidium iodide (staining only dead cells) and membrane permeable Hoechst 33342 (staining live and dead cells), and the percent apoptosis was determined by quantifying the portion of propidium iodide stained cells in the total population. Propranolol treatment resulted in a significantly increased apoptosis in each cell line, with no propidium iodide cells detected in any of the sham treatments **(** **Figure 4A & B** **)**. Nuclear modeling of Hoechst 33342 stained SVR cells treated with 100 µM propranolol revealed nuclear morphology alterations indicative of apoptosis **(** **Figure 4C** **)**, and given that pathway analysis of our microarray data revealed a strong apoptotic node, we quantified the steady state mRNA expression levels of 84 genes involved in apoptosis using SVR cells treated for 24 hours with sham or 100 µM propranolol. We identified 18 genes whose expression was significantly altered 1.5 fold or more (p<0.05) **(** **Figure 4D** **)**, indicating that beta blockade regulates the network of genes controlling angiosarcoma cell survival. Furthermore, staining for cleaved-caspase 3 was observed in SVR angiosarcoma cells treated for 24 hours with 100 µM propranolol, but undetectable in sham conditions **(** **Figure 4E** **)**. 1 hour of propranolol treatment in SVR cells resulted in marked increases in the phosphorylation of p38 MAPK **(** **Figure 4F** **)**, suggesting that cells are rapidly responding by initiating the stress response pathway. No changes in the phosphorylation of p42/44 or JNK/SAPK were detected following propranolol treatment *(data not shown)*. After 24 hours, Western analysis revealed that 100 µM propranolol treatment of SVR cells resulted in increased p53, cleaved caspase 3 and cleaved PARP **(** **Figure 4G** **)**, indicative of an apoptotic response. Despite propranolol’s ability to induce apoptosis at relatively higher concentrations, this effect must be selective for tumor cells, yet spare normal cells. We previously reported that primary cultures of human endothelial cells exhibit reduced proliferation at 50 µM propranolol, however no apoptosis was observed at propranolol concentrations less than 150 µM [26]. To test if propranolol exhibits selectivity against malignant vascular tumor cells, we performed co-culture experiments where unstained SVR cells were grown in the presence of CellTracker Blue dye stained human dermal microvascular endothelial cells (HDMVECs). After 48 hours, sham treatment resulted in a confluent layer of SVR cells intermixed with dispersed HDMVECs cells **(** **Figure 4H** **)**. In contrast, 100 µM propranolol resulted in complete detachment of SVR cells, with only labeled HDMVECs attached to the substrate.

**Figure 5:**
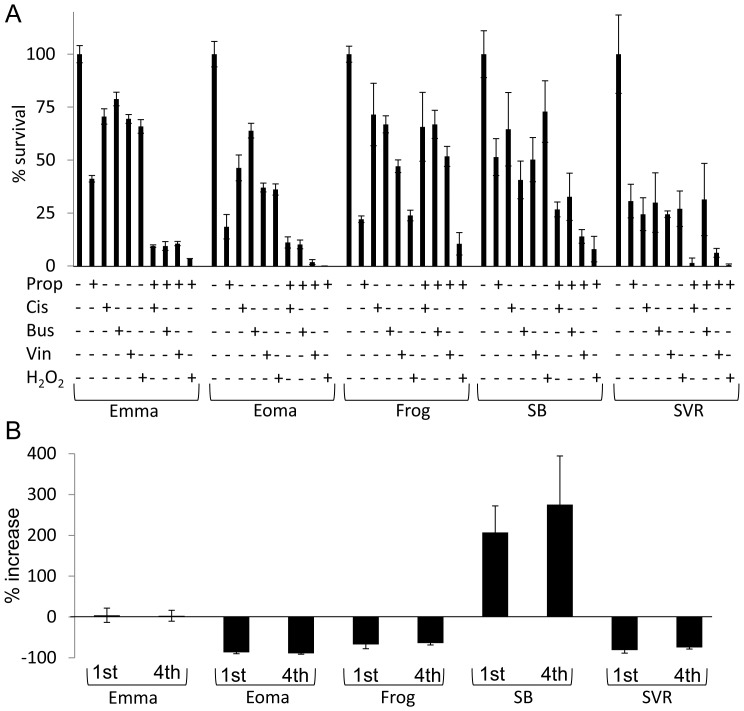
Beta blockade synergizes with chemotherapy to induce apoptosis of malignant vascular tumor cells and does not result in accumulation of chemoresistance. (**A**) The panel of malignant vascular tumor cells were treated with 100 µM propranolol, cisplatin (cis), busulfan (bus), vincristine (vin), or H_2_O_2_ alone or in combination and cell survival was assessed after 24 hours. (**B**) The cell panel was treated with 100 µM propranolol until approximately 5% of the culture remained alive. The remaining cells were allowed to repopulate the culture and this process was repeated three subsequent times. Cell survival was assessed after 24 hours of 100 µM propranolol treatment of the naïve (1^st^) tumor cells and selected (4^th^) tumor cells. For all experiments, the data is the average +/− standard deviation for at least three biological replicates. Statistical significant was determined using Students t-test (p<0.05).

Even following chemotherapy and radiation, patients with angiosarcomas and metastatic hemangioendotheliomas experience a high mortality rate. If beta blockade is ever used in a clinical setting, it will likely be used in combination with standard anti-angiosarcoma chemotherapeutics, thus we tested if propranolol is effective as a combination therapeutic against malignant vascular tumors. We treated the cell panel with sham, 100 µM propranolol, chemotherapeutic/cytotoxic agents (cisplatin, busulfan, vincristine, or H_2_O_2_ induced free radical damage) alone, or the combination of propranolol and these agents. Either propranolol or the toxic agents led to significant reductions in cell survival after 48 hours in all cell lines, however the combination of propranolol plus the chemotherapeutic/cytotoxic agents resulted in synergistic reductions in survival for all lines except Frog **(** **Figure 5A** **)**. One of the most common reasons for mortality in cancer patients is acquisition of drug resistance following treatment, therefore we tested if our cell panel is capable of developing resistance to propranolol. The cells were treated with lethal doses (100 µM) of propranolol for several days and the few surviving cells were allowed to repopulate the culture. This process was repeated multiple times. Cell survival was measured between naïve cells (*1^st^ treatment*) and cells after four rounds of propranolol selection (*4^th^ treatment*). Cell survival was statistically no different between naïve cells and experienced cells **(** **Figure 5B** **)**, suggesting that resistance to propranolol may be minimal. This data suggests that propranolol may enhance the efficacy of standard therapies without increasing tumor resistance.

### Propranolol Disrupts the Migration of Malignant Vascular Tumors

**Figure 6:**
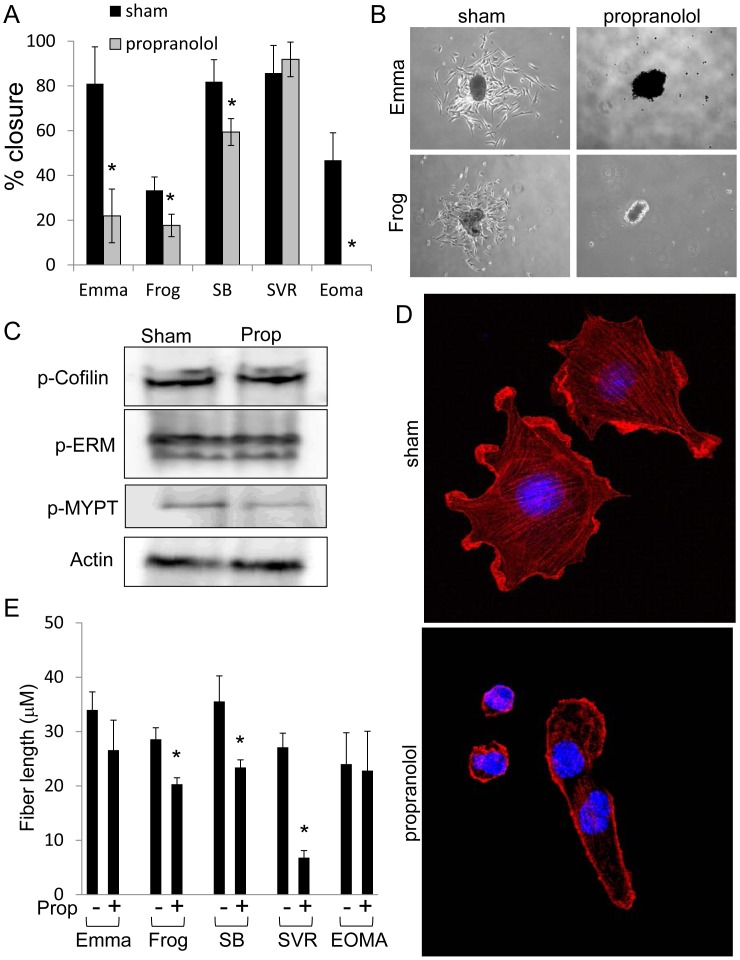
Beta blockade inhibits migration in malignant vascular tumor cells. (**A**) The panel of malignant vascular tumor lines was subjected to a scratch assay in the presence of sham or 50 µM propranolol. The percent closure of the wound was assessed after 18 hours. (**B**) Emma and Frog cells were grown as tumor spheroids. Spheroids were allowed to attach to a substrate and to migrate out from the central mass in the presence of sham or 50 µM propranolol for 24 hours. (**C**) Western detection of cytoskeletal regulators activation. (**D**) SVR cells were treated with sham or 50 µM propranolol for 24 hours and labeled with rhodamine-conjugated phalloidin and Hoechst. (**E**) Computational FiberScore analysis quantifying the mean actin stress fiber length in sham or 50 µM propranolol treated cells. For all experiments, the data is the average +/− standard deviation for at least three biological replicates. Statistical significant was determined using Students t-test (p<0.05).

To test if propranolol affects malignant vascular tumor cell migration, we performed scratch assays on our cell panel. To avoid cell death from high doses of propranolol, we reduced the concentration to 50 µM, for which no net negative decreases in cell proliferation were observed for any line **(** **Figure 2A** **)**. At this dosage, we were able to accurately measure migration changes without changes in cell death complicating the data analysis. For all lines tested except SVR, 50 µM propranolol reduced closure of the wound relative to the sham **(** **Figure 6A** **)**. To corroborate these findings, we employed a 3D growth assay where cells were cultured as 3D spheres, plated onto a substrate in the presence of 50 µM propranolol or sham, and allowed to invade into the adjacent surroundings over 24 hours. Similar to our reasoning with scratch wounding migration assays, we used a sublethal dose of propranolol (50 µM) to evaluate migration independent of cell death. Only Emma and Frog assembled into well formed tumor spheroids, thus we proceeded with using these lines. The sham treated tumor spheroids aggressively migrated from the spheroid mass into surrounding areas over the 24 hour period, while 50 µM propranolol abolished all migration (**Figure 6B** **)**. To understand the mechanism by which propranolol inhibits cell movement and invasion, we first tested the phosphorylation status of key cytoskeletal regulators in SVR angiosarcoma cells. We have previously shown that propranolol treatment of infantile hemangioma endothelial cells decreased phosphorylation of the actin regulatory protein cofilin [26], however Western blot analysis of SVR cells showed no change in the phosphorylation of cofilin, ezrin/radixin/moesin (ERM), or myosin phosphatase targeting subunit (MYPT) in reponse to propranolol **(** **Figure 6C** **)**. Imaging of actin microfilaments revealed that sham SVR cells exhibited well developed actin stress fibers and numerous lamellipodia, while sublethal doses of propranolol (50 µM) resulted in dissolution of central actin stress fibers, with only cortical actin remaining along the cell periphery **(** **Figure 6D** **)**. We measured the median stress fiber length of sham or 50 µM propranolol treated malignant vascular tumor cells using the FiberScore algorithm [27], revealing a significantly marked reduction in stress fiber length following propranolol treatment in all but the Emma and EOMA lines **(** **Figure 6E** **)**.

### Propranolol Inhibits Angiosarcoma Tumor Growth

**Figure 7:**
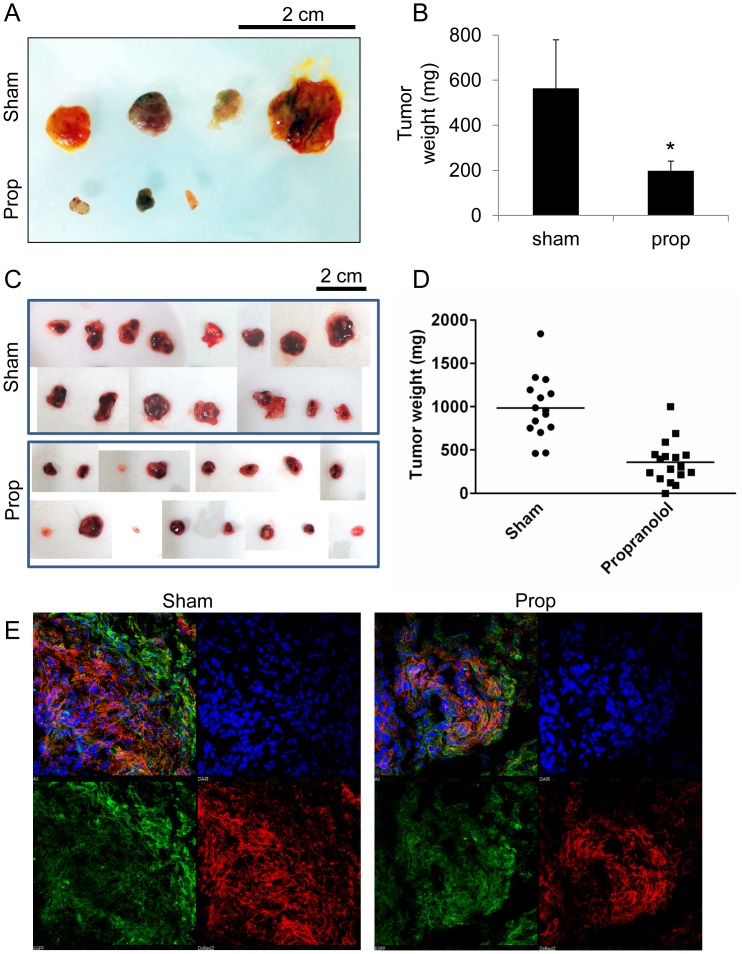
Beta blockade exhibits efficacy as a single agent therapy against *in vivo* angiosarcoma tumors. (**A**) SVR cells were implanted onto the CAM of chick embryos. The tumors were treated every 48 hours with a 100 µM propranolol or sham and harvested after 6 days treatment. (**B**) Weight of the SVR tumors collected from the CAM assay. The data is the average +/− standard. Statistical significant was determined using Students t-test (p<0.05). (**C**) SVR cells were injected subcutaneously into the dorsolateral flanks of 4 week old host mice. Mice were injected with sham or 10 mg/kg propranolol via intraperitoneal route every two days. (**D**) Weight of the SVR angiosarcoma tumors collected from the mouse angiosarcoma experiment. All tumor weights are included in the graph, and the horizontal bar represents the mean tumor weight. Statistical significant was determined using Students t-test (p<0.05). (**E**) Immunofluorescent staining for cell proliferation (PCNA protein = green), actin cytoskeleton (*red*), and cell number (*DAPI = blue*) of frozen sections collected from sham or propranolol SVR tumors isolated from the mouse tumor experiment.

To move our findings into more relevant *in vivo* models, we employed the chorioallantoic membrane (CAM) tumor assay where SVR cells in a gelatin sponge were placed onto the vascularized CAM of 8 day post-fertilization chicken eggs. Tumors were treated with a sham or 100 µM propranolol every two days over a six day time course, after which the tumors were excised, revealing significant reductions in tumor size and weight in propranolol treated tumors **(** **Figure 7A & B** **)**. We expanded our studies into an established mouse angiosarcoma tumor model [29] where the animals were systemically treated with saline sham or 10 mg/kg propranolol every 2 days. Tumor collection occurred when the sham tumors reached approximately 1 cm^3^. Propranolol therapy resulted in significantly reduced angiosarcoma tumor size and weight (357+/−58 mg; N = 17, p<0.0001) vs sham (984+/−92 mg; N = 15, p<0.0001) **(** **Figure 7C & D** **)**. Despite the reduction in tumor size following propranolol administration, tumor sections from both sham and propranolol conditions revealed PCNA expression at the tumor peripheries, indicating active cell division **(** **Figure 7E** **)**. This suggests that future studies should employ combinatorial therapy with propranolol.

## Discussion

Malignant vascular tumors such as angiosarcomas and metastatic hemangioendotheliomas are rare solid tumors that readily metastasize due to their intrinsic vascular nature. These tumors are exceptionally difficult to treat and several phase II trials investigating the therapeutic efficacy of novel anti-angiogenic drugs such as Bevacizumab, Sunitinib, and Sorafenib have resulted in a minimal to absent response in vascular tumor patients [30], [31], [32]. Of the three drugs, Sorafenib exhibited an angiosarcoma subtype-specific response in 14% of patients, however the median progression free survival was only 3.8 months, with 81% of angiosarcoma patients and 100% of hemangioendothelioma patients exhibiting tumor progression after 6 months of treatment [32]. This indicates the strong need to develop more effective therapies against malignant vascular tumors.

Given that we **(** **Figure 1A–C** **)** and others have demonstrated that malignant vascular tumors express high levels of beta adrenergic receptors [15], [26], we hypothesized that beta blockade might demonstrate therapeutic efficacy against these tumors. We reveal that propranolol selectively inhibits proliferation, survival, and migration of a panel of malignant vascular tumor cells, indicating that the oncogenic properties of these tumor types are driven, in part, by beta adrenergic signaling. While propranolol dramatically slows the proliferation rate of all vascular tumor lines tested, four of the five tumor lines exhibited nearly 100% lethality at doses that we have previously reported to be non-toxic for primary cultures of human endothelial cells **(** **Figure 2A** **)** [26]. Indeed, co-culture of HDMVECs and angiosarcoma cells in the presence of propranolol results in selective killing of the angiosarcoma cells **(** **Figure 4H** **)**. These findings indicate that malignant vascular tumor lines are exquisitely sensitive to beta blockade and provide strong support for its use in animal and human models. It should be noted that while beta blockade was effective in reducing the proliferation rate and increasing cellular apoptosis for all cell lines, there were varying levels of resistance between the lines. For instance, the SB line exhibited high resistance to propranolol-induced alterations in proliferation and apoptosis **(** **Figure 2A** **,** **Figure 5A & B** **)**, while the EOMA hemangioendothelioma cell line consistently showed the highest susceptibility to beta blockade. Future studies should elucidate the molecular profiles characteristic of each of these lines that might serve as putative biomarkers of susceptibility to propranolol.

Our data demonstrate that propranolol blocks proliferation and survival in part through altering levels of key cell cycle and apoptotic regulators **(** **Figures 3** **&** **4** **)**. Our data indicates that propranolol induces a strong upregulation of *Tek*. *Tek* is preferentially expressed on endothelial cells, encodes for the Tie2 angiogenic receptor, and strongly promotes vessel regression, quiescence, and stability in a context dependent manner [33]. We suspect that in addition to general cell cycle alterations, propranolol’s specificity for vascular tumors may function through Tie2-mediated modulation of vessel stability and maturation, however the Ang/Tie2 network is a very complex signaling pathway interdependent on a number of other proteins, thus future work should address this issue.

Using two *in vivo* tumor models we demonstrated efficacy for propranolol as an anti-angiosarcoma agent. However, propranolol as a stand-alone therapeutic (like most therapeutics) did not completely abolish tumor cell proliferation as evidenced by positive PCNA staining in propranolol treated tumors. Given our exciting data showing synergistic effects on malignant vascular tumor lines treated with combined propranolol and chemotherapy **(** **Figure 5A** **)**, future emphasis should be placed on determining the optimal strategy for beta blockade against hemangioendotheliomas and angiosarcomas. Indeed, a recent publication reported propranolol potentiates the anti-tumor effects of chemotherapy in preclinical models of breast cancer [10]. Such an endeavor should not prove difficult given that propranolol has been used extensively to treat hypertension, anxiety, and post-traumatic stress syndrome since its Nobel Prize winning discovery in the 1960’s, and its toxicity and side effects are well established in pediatric and adult patients.

In this study, we employ a novel application of beta blockade for the successful treatment of malignant vascular tumors using *in vitro* and *in vivo* models. These findings are significant because metastatic hemangioendotheliomas and angiosarcomas are very rare aggressive tumor types that are markedly understudied, exceptionally difficult to treat, and result in high patient mortality. These data lay the groundwork for future clinical trials to test the off-label use of the generic drug propranolol for a population of patients in desperate need of effective tumor treatments.
